# Supplementary material for: Economic use of plants is key to their naturalization success
Source: Nat Commun. 2020 Jun 24;11:3201. doi: 10.1038/s41467-020-16982-3 (PMC7314777; doi:10.1038/s41467-020-16982-3)
Supplement: Supplementary file 1 — Supplementary Information [file 41467_2020_16982_MOESM1_ESM.pdf]

Economic use of plants is key to their naturalization success

van Kleunen et al.

Supplementary Table 1 | Results of phylogenetic binomial GLM (with logit link) testing the effects of each economic use on naturalization success. The analysis included all seed plants (n=326,101). Significance of the coefficients was determined with two-sided z-tests without correction for multiple comparisons. The phylogenetic correlation parameter α is 0.204. The model coefficients were back-logit-transformed to calculate the naturalization probabilities shown in Fig. 2c.

Model term	Estimate	SE	z-value	p
Intercept (no use)	-4.051	0.019	-210.13	<0.001
Animal food	2.930	0.102	28.81	<0.001
Bee plants	1.492	0.238	6.27	<0.001
Environmental	3.586	0.036	100.97	<0.001
Food additives	0.989	0.140	7.08	<0.001
Fuels	0.178	0.234	0.76	0.447
Gene sources	1.592	0.071	22.35	<0.001
Human food	1.680	0.084	20.00	<0.001
Invertebrate food	0.591	0.481	1.23	0.220
Materials	1.029	0.070	14.61	<0.001
Medicines	2.288	0.057	40.09	<0.001
Non-vertebrate poisons	0.956	0.436	2.19	0.028
Social	-0.087	0.271	-0.32	0.749

Supplementary Table 2 | Results of binomial GLM (with logit link) testing the effects of each economic use on naturalization success. The analysis included all seed plants (n=326,101). Significance of the coefficients was determined with two-sided z-tests without correction for multiple comparisons. The model coefficients were back-logit-transformed to calculate the naturalization probabilities shown in Supplementary Fig. 5.

Model term	Estimate	SE	z-value	p
Intercept (no use)	-3.685	0.011	-323.23	<0.001
Animal food	3.025	0.097	31.24	<0.001
Bee plants	1.486	0.230	6.47	<0.001
Environmental	3.161	0.033	95.78	<0.001
Food additives	0.939	0.136	6.90	<0.001
Fuels	0.157	0.224	0.70	0.484
Gene sources	1.428	0.061	23.38	<0.001
Human food	1.627	0.081	20.17	<0.001
Invertebrate food	0.587	0.467	1.26	0.209
Materials	0.795	0.068	11.65	<0.001
Medicines	2.197	0.053	41.26	<0.001
Non-vertebrate poisons	0.953	0.430	2.22	0.027
Social	-0.098	0.260	-0.38	0.707

Supplementary Table 3 | Results of phylogenetic binomial GLM (with logit link) testing the effects of specific combinations of two economic uses on naturalization success. The analysis included all seed plants with no, one or two economic uses (n= 324,875). Significance of the coefficients was determined with two-sided z-tests without correction for multiple comparisons. The phylogenetic correlation parameter α is 0.202. The model coefficients were used to make Fig. 4.

Model term	Estimate	SE	z-value	p
Intercept	-4.209	0.021	-197.67	<0.001
Animal food	4.025	0.115	35.01	<0.001
Bee plants	3.276	0.483	6.79	<0.001
Environmental	4.004	0.040	99.39	<0.001
Food additives	2.850	0.332	8.60	<0.001
Fuels	1.585	1.251	1.27	0.205
Gene sources	2.185	0.080	27.31	<0.001
Human food	3.246	0.120	27.13	<0.001
Invertebrate food	-8.812	475.340	-0.02	0.985
Materials	2.352	0.102	22.97	<0.001
Medicines	3.206	0.065	49.14	<0.001
Non-vertebrate poisons	-8.817	389.017	-0.02	0.982
Social	1.139	1.247	0.91	0.361
Animal food × Environmental	-2.289	0.219	-10.45	<0.001
Animal food × Gene sources	-1.840	0.335	-5.50	<0.001
Animal food × Human food	-2.889	0.543	-5.33	<0.001
Animal food × Materials	-3.692	0.817	-4.52	<0.001
Animal food × Medicines	-1.533	0.756	-2.03	0.042
Bee plants × Environmental	-2.186	0.624	-3.50	<0.001
Bee plants × Materials	-1.390	0.779	-1.79	0.074
Environmental × Food additives	-1.384	0.747	-1.85	0.064
Environmental × Fuels	-0.965	1.408	-0.69	0.493
Environmental × Gene sources	-1.655	0.158	-10.46	<0.001
Environmental × Human food	-2.442	0.229	-10.67	<0.001
Environmental × Materials	-1.708	0.155	-11.01	<0.001
Environmental × Medicines	-1.926	0.127	-15.12	<0.001
Food additives × Materials	-2.375	0.745	-3.19	0.001
Food additives × Medicines	-1.911	0.412	-4.64	<0.001
Fuels × Materials	-0.145	1.331	-0.11	0.913
Gene sources × Human food	-1.483	0.238	-6.23	<0.001
Gene sources × Materials	-0.742	0.559	-1.33	0.185
Gene sources × Medicines	-1.238	0.411	-3.01	0.003
Human food × Materials	-2.805	0.319	-8.78	<0.001
Human food × Medicines	-1.461	0.257	-5.69	<0.001
Materials × Medicines	-1.772	0.201	-8.82	<0.001
Medicines × Social	-1.636	1.414	-1.16	0.247

Supplementary Table 4 | Results of binomial GLM (with logit link) testing the effects of specific combinations of two economic uses on naturalization success. The analysis included all seed plants with no, one or two economic uses (n=324,875). Significance of the coefficients was determined with two-sided z-tests without correction for multiple comparisons.

Model term	Estimate	SE	z-value	p
Intercept	-3.750	0.012	-315.02	<0.001
Animal food	3.994	0.114	34.93	<0.001
Bee plants	3.265	0.450	7.26	<0.001
Environmental	3.569	0.037	97.40	<0.001
Food additives	2.770	0.303	9.14	<0.001
Fuels	1.553	1.054	1.47	0.141
Gene sources	1.846	0.068	27.08	<0.001
Human food	3.049	0.113	26.93	<0.001
Invertebrate food	-8.816	229.628	-0.04	0.969
Materials	2.065	0.096	21.54	<0.001
Medicines	2.898	0.062	47.08	<0.001
Non-vertebrate poisons	-8.816	187.491	-0.05	0.962
Social	1.111	1.035	1.07	0.283
Animal food × Environmental	-2.278	0.219	-10.40	<0.001
Animal food × Gene sources	-1.856	0.334	-5.55	<0.001
Animal food × Human food	-2.887	0.551	-5.24	<0.001
Animal food × Materials	-3.694	0.804	-4.59	<0.001
Animal food × Medicines	-1.532	0.785	-1.95	0.051
Bee plants × Environmental	-2.190	0.600	-3.65	<0.001
Bee plants × Materials	-1.397	0.760	-1.84	0.066
Environmental × Food additives	-1.385	0.725	-1.91	0.056
Environmental × Fuels	-0.967	1.237	-0.78	0.434
Environmental × Gene sources	-1.665	0.152	-10.96	<0.001
Environmental × Human food	-2.455	0.222	-11.05	<0.001
Environmental × Materials	-1.723	0.149	-11.53	<0.001
Environmental × Medicines	-1.936	0.121	-16.07	<0.001
Food additives × Materials	-2.383	0.725	-3.29	0.001
Food additives × Medicines	-1.945	0.390	-4.99	<0.001
Fuels × Materials	-0.155	1.147	-0.14	0.892
Gene sources × Human food	-1.512	0.234	-6.45	<0.001
Gene sources × Materials	-0.748	0.570	-1.31	0.189
Gene sources × Medicines	-1.235	0.413	-2.99	0.003
Human food × Materials	-2.829	0.322	-8.78	<0.001
Human food × Medicines	-1.488	0.251	-5.92	<0.001
Materials × Medicines	-1.794	0.200	-8.95	<0.001
Medicines × Social	-1.645	1.221	-1.35	0.178

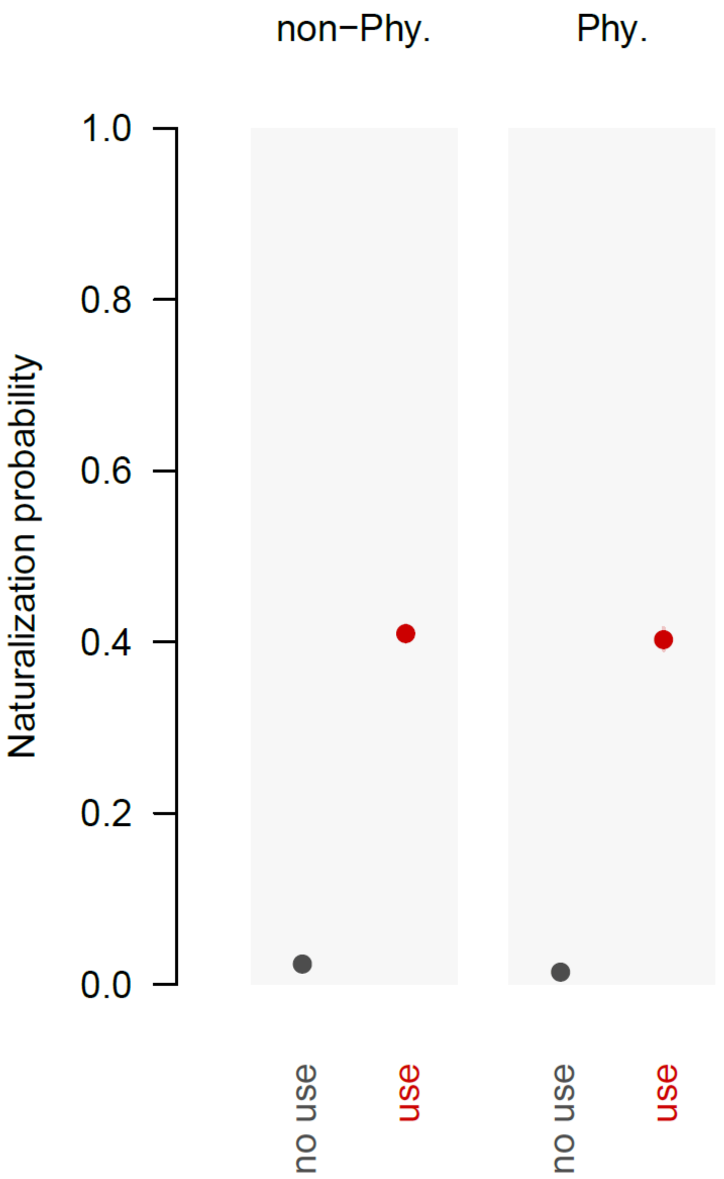

Supplementary Fig. 1 | Naturalization probability of plants without and with known economic use in the extant global seed-plant flora. The left panel shows the results based on a binomial GLM, and the right panel shows the results based on a phylogenetic binomial GLM. These are alternative analyses for the resampling test that accompanied Fig. 1.

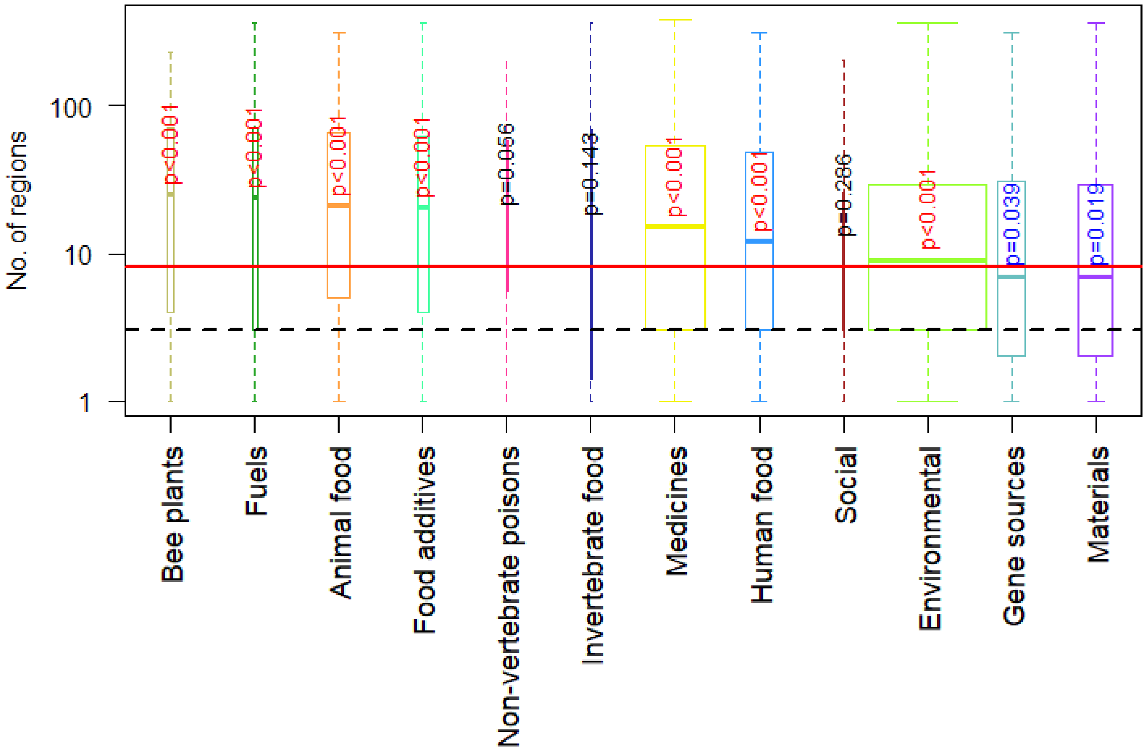

Supplementary Fig. 2 | Naturalization extent of naturalized taxa with economic uses. The boxplots show the number of GloNAF regions in which species have become naturalized for the subset of naturalized taxa (n=4,792) in each of the different economic use categories. The width of each boxplot is proportional to the number of taxa. The economic use categories are ranked according to the median extent of naturalization. The red horizontal line indicates the median number (8) of GloNAF regions for naturalized taxa in the WEP dataset. The black dashed line indicates the median number (3) of GloNAF regions of all naturalized taxa. P-values from two-sided resampling tests indicate whether the proportion of naturalized taxa is significantly higher (red) or lower (blue) than expected or does not deviate from expectations (black). No corrections were made for multiple comparisons. The boxes indicate the interquartile range, the fat horizontal line indicates the median and the whiskers indicate the range of values.

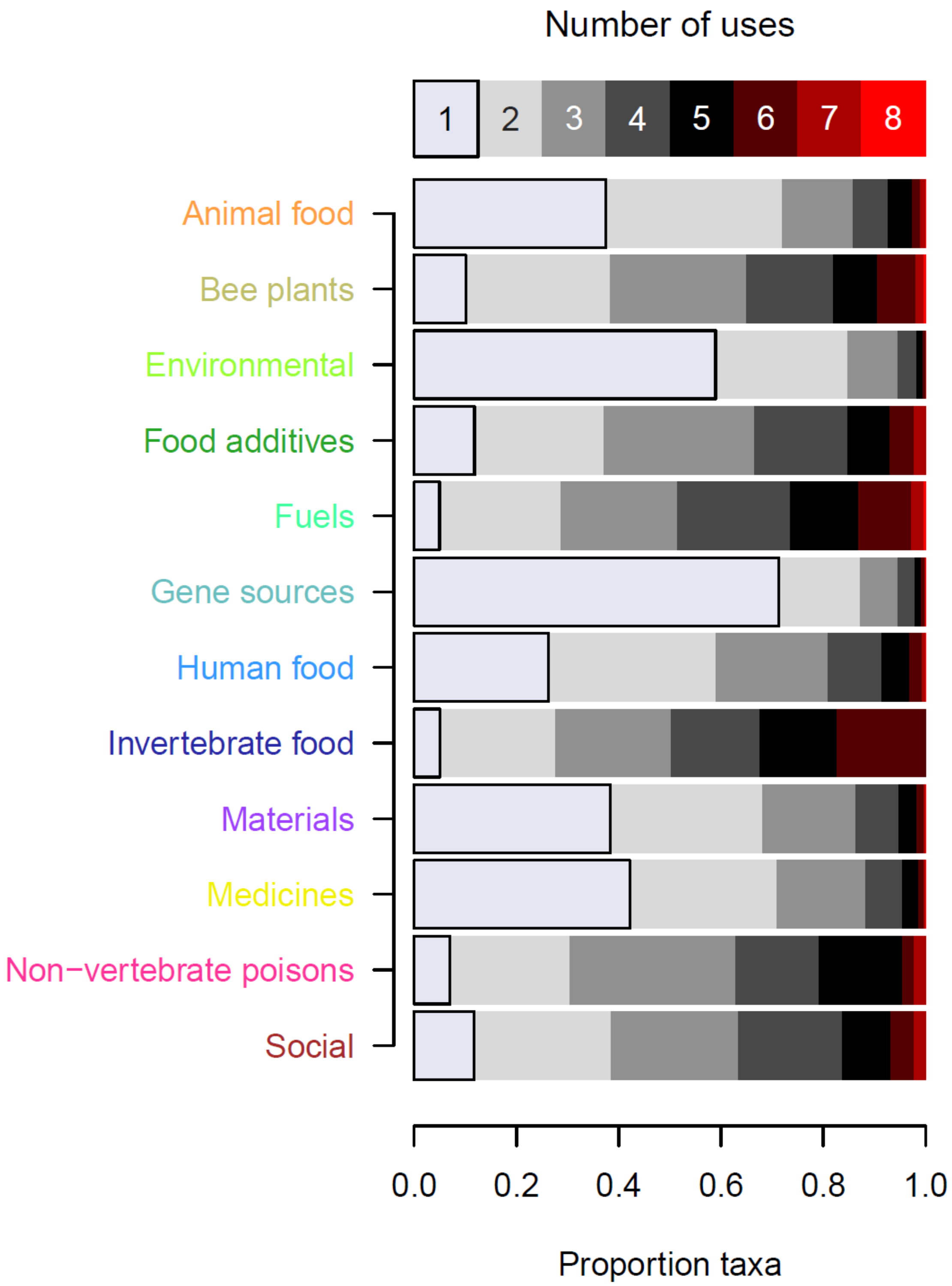

Supplementary Fig. 3 | Stacked bar plot showing for each focal economic use the proportion of taxa that have one or multiple (2, 3, 4, 5, 6, 7, 8) economic uses.

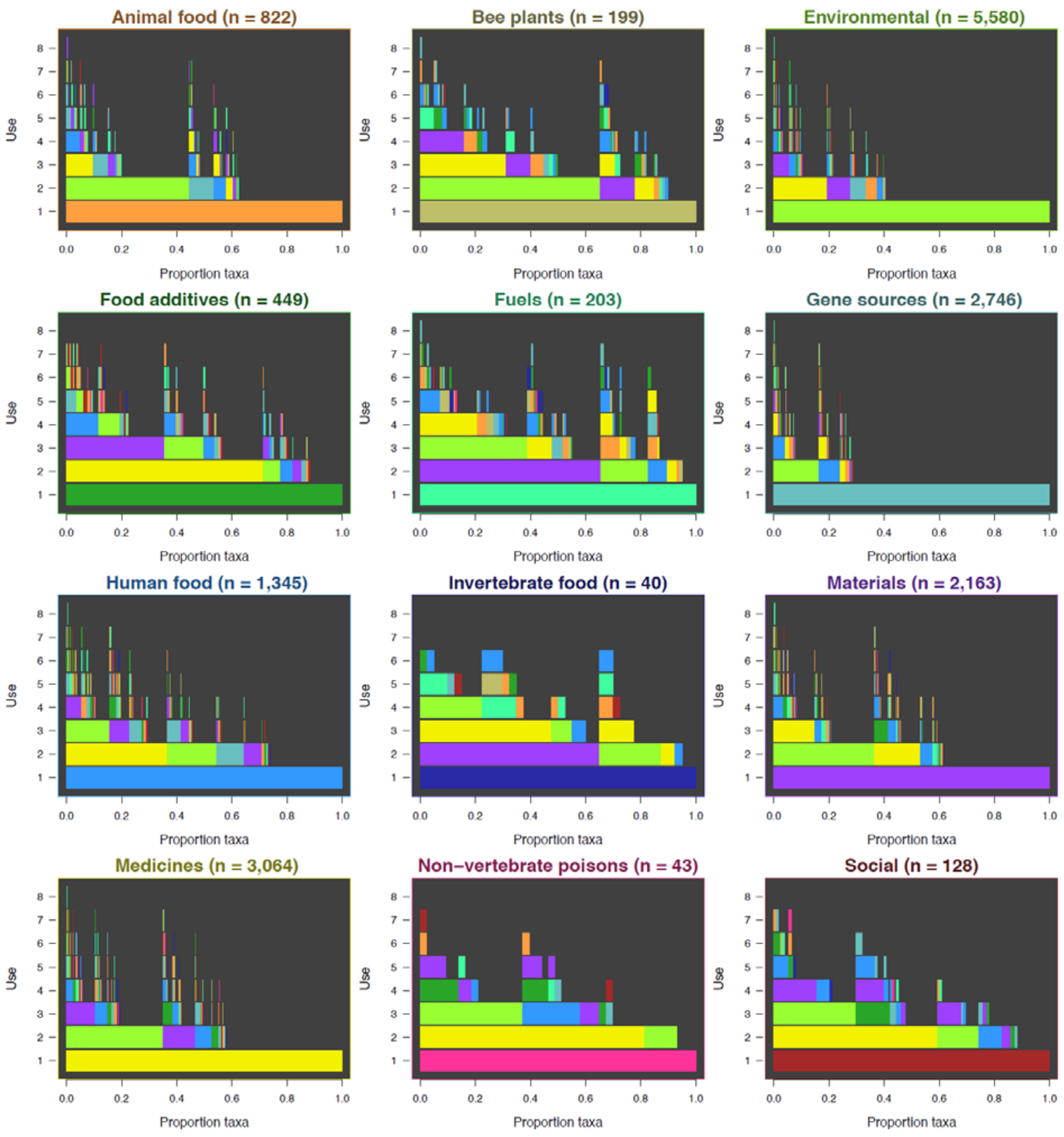

Supplementary Fig. 4 | Proportions of taxa with each possible combination of a focal economic use with the other economic uses. The color scheme for the economic uses corresponds to the one used in Fig. 2 of the main manuscript, and to the color of the bottom bar in each panel (e.g. animal food is orange).

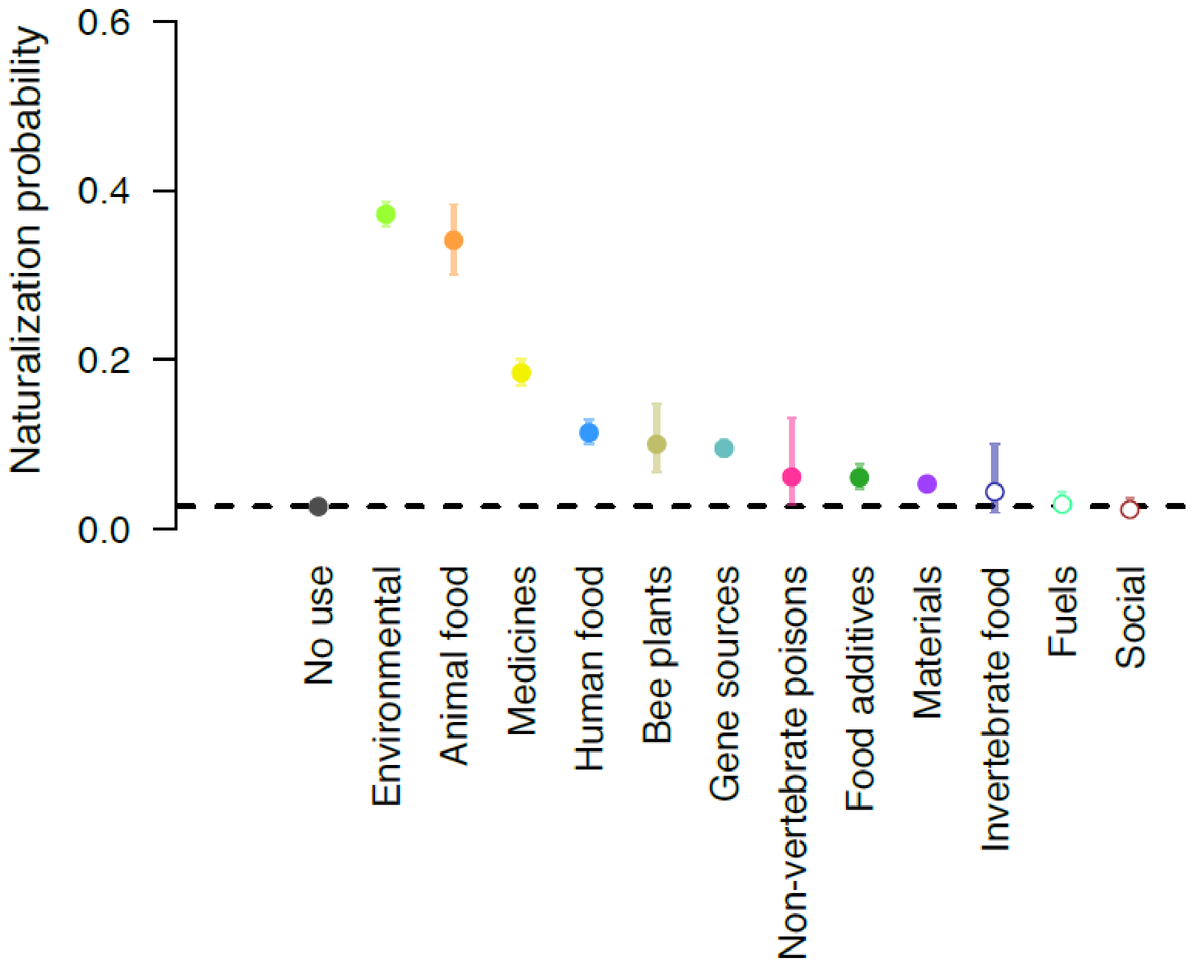

Supplementary Fig. 5 | Naturalization probability of plants within different economic use categories compared to plants without a known economic use. The figure shows the results based on a binomial GLM analysing economic uses of all seed plants (n=326,101). Error bars indicate 95% confidence intervals. Open circles indicate economic uses for which the naturalization probability does not differ significantly from taxa with no economic uses ($p<0.05$; for exact p-values see Supplementary Table 2). This is the corresponding non-phylogenetic version of the results of the phylogenetic binomial GLM shown in Fig. 2c.

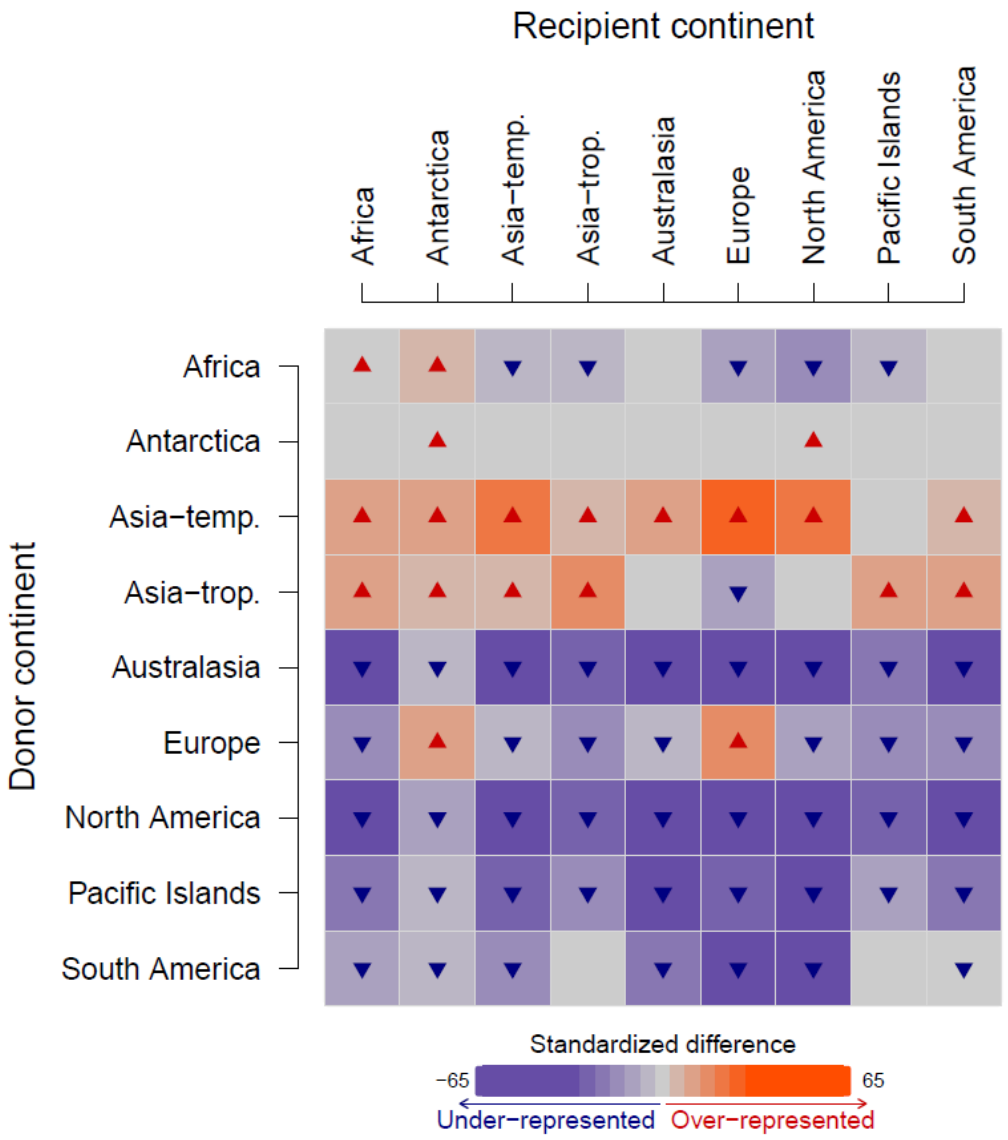

Supplementary Fig. 6 | Flows of naturalized economic plants among the TDWG continents. Standardized difference between observed and expected numbers of naturalized economic plants from each donor TDWG continent in each of the recipient TDWG continents. The standardized difference is the difference between the observed flow and the mean of the expected flows (based on 9,999 random draws form the WEP dataset taxa) divided by the standard deviation of the expected flows. We used two-sided resampling tests to determine which flows deviated significantly ($p<0.05$) from expectations. No corrections were made for multiple comparisons. Flows that are significantly larger than expected have a red upward-pointing triangle, and flows that are significantly smaller than expected have a blue downward pointing triangle.

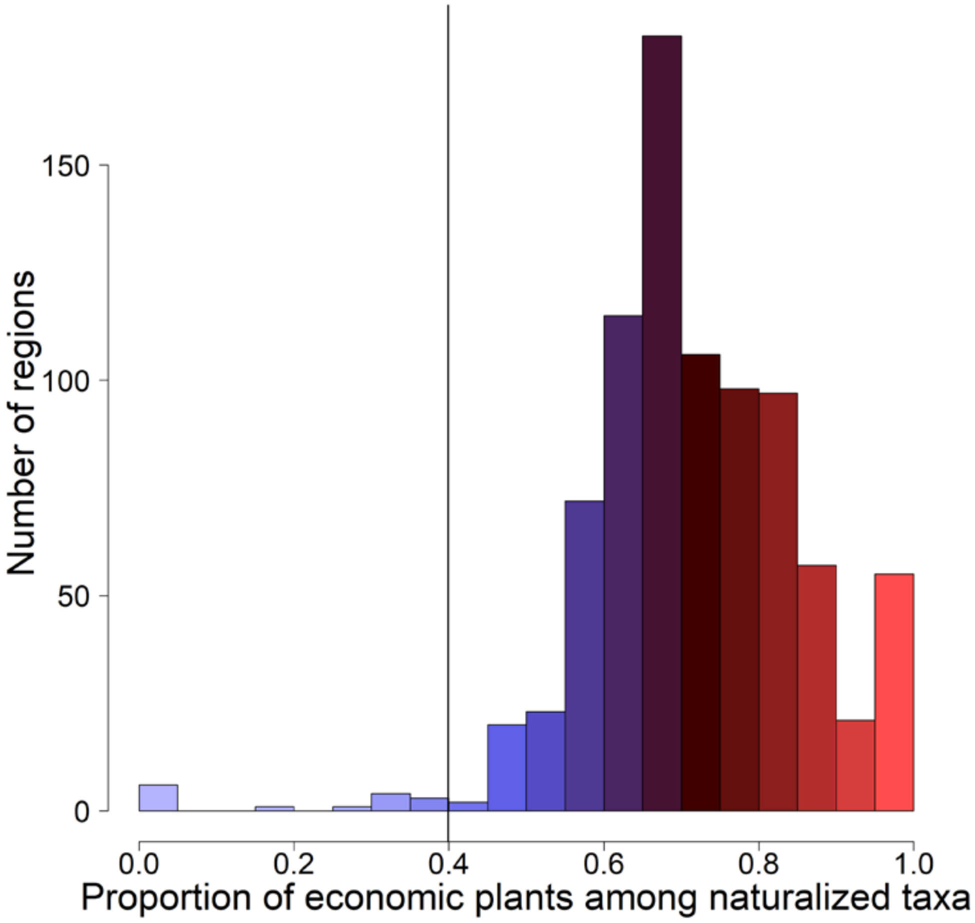

Supplementary Fig. 7 | Histogram of the proportion of economic plants among naturalized taxa in the GloNAF regions. The black vertical line indicates the proportion of economic plants in the global alien flora. The different colors correspond to the ones used in the map of Fig. 7a.

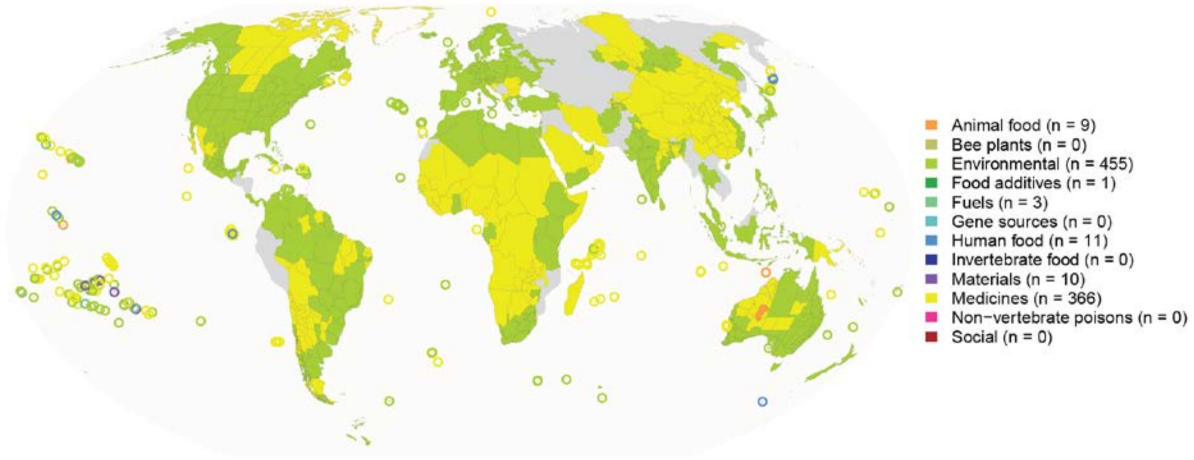

Supplementary Fig. 8 | Map of the world showing for each of the GloNAF regions the dominant economic use among the naturalized economic plants. Circles were used to increase the visibility of small islands and island groups on the map. The numbers behind each economic use in the legend indicate the number of regions where this economic use is dominant among the naturalized economic plants.

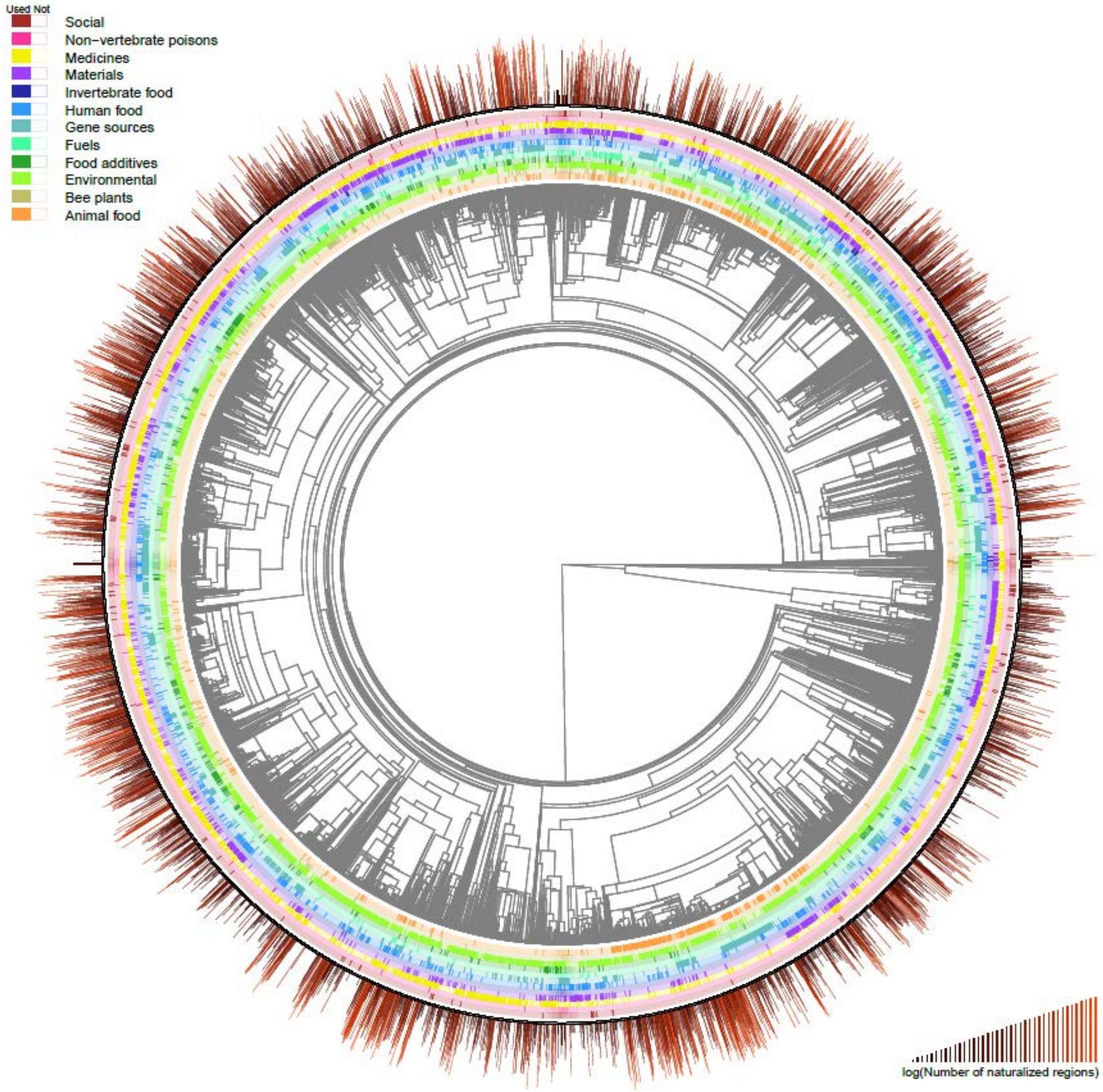

Supplementary Fig. 9 | Phylogenetic tree of the economic plants in the WEP dataset. The distribution of the different economic uses is shown in concentric rings around the tips, and the naturalization extent (number of GloNAF regions) is shown as bars in the outer ring.

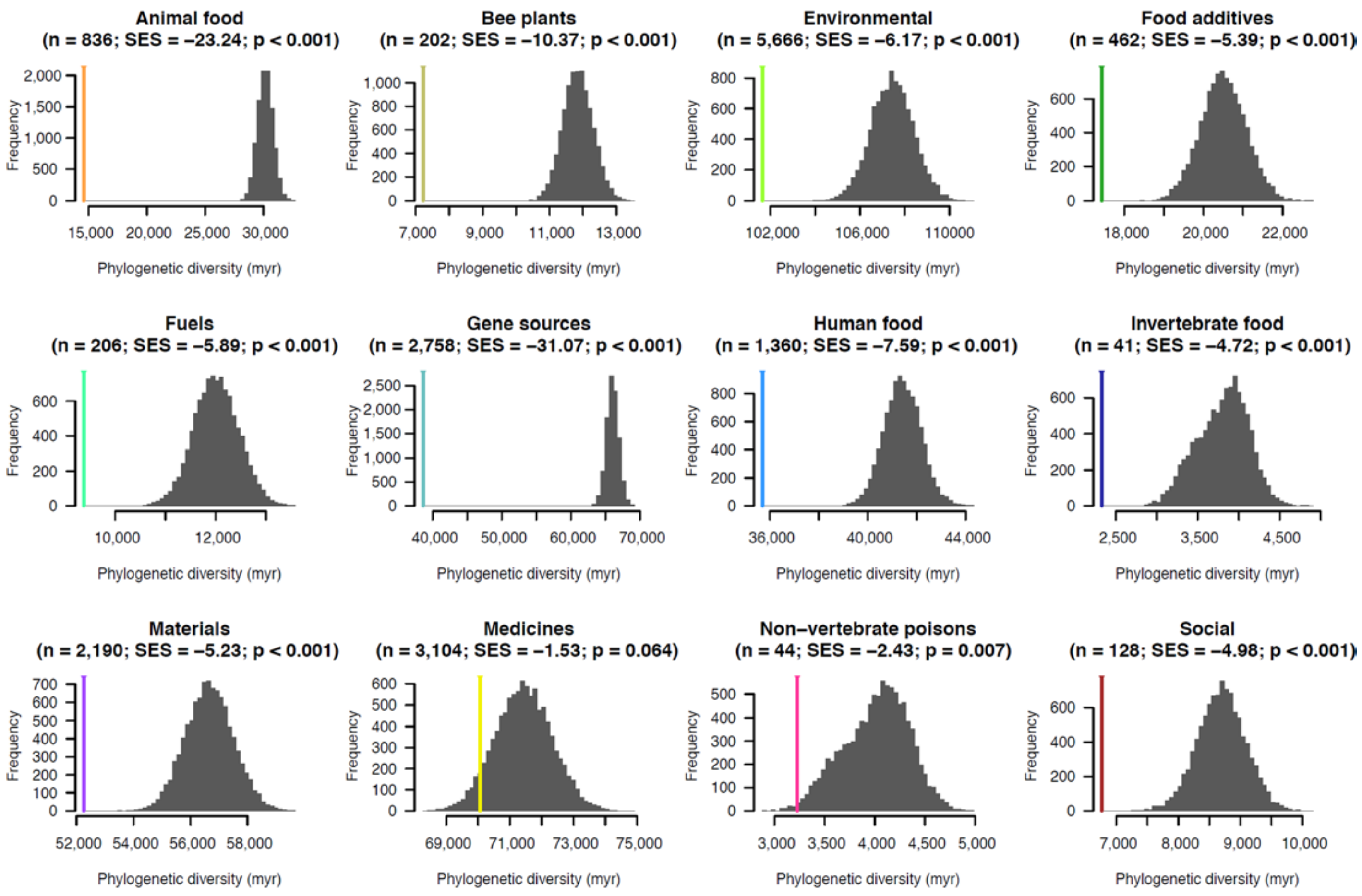

Supplementary Fig. 10 | Test for phylogenetic structure of each economic use category among all economic plants in the WEP dataset. The colored lines indicate the observed phylogenetic diversity (in million years, myr) encompassed by plants of the particular economic use, and the histograms indicate the expected phylogenetic diversities based on random sampling from all economic plants in the WEP dataset (n=11,685). We used two-sided resampling tests to determine observed phylogenetic diversity values deviated significantly ($p<0.05$) from expectations. No corrections were made for multiple comparisons. If the observed phylogenetic diversity is in or below the lower 2.5% of the distribution of expected values, the phylogenetic structure is significantly clustered.

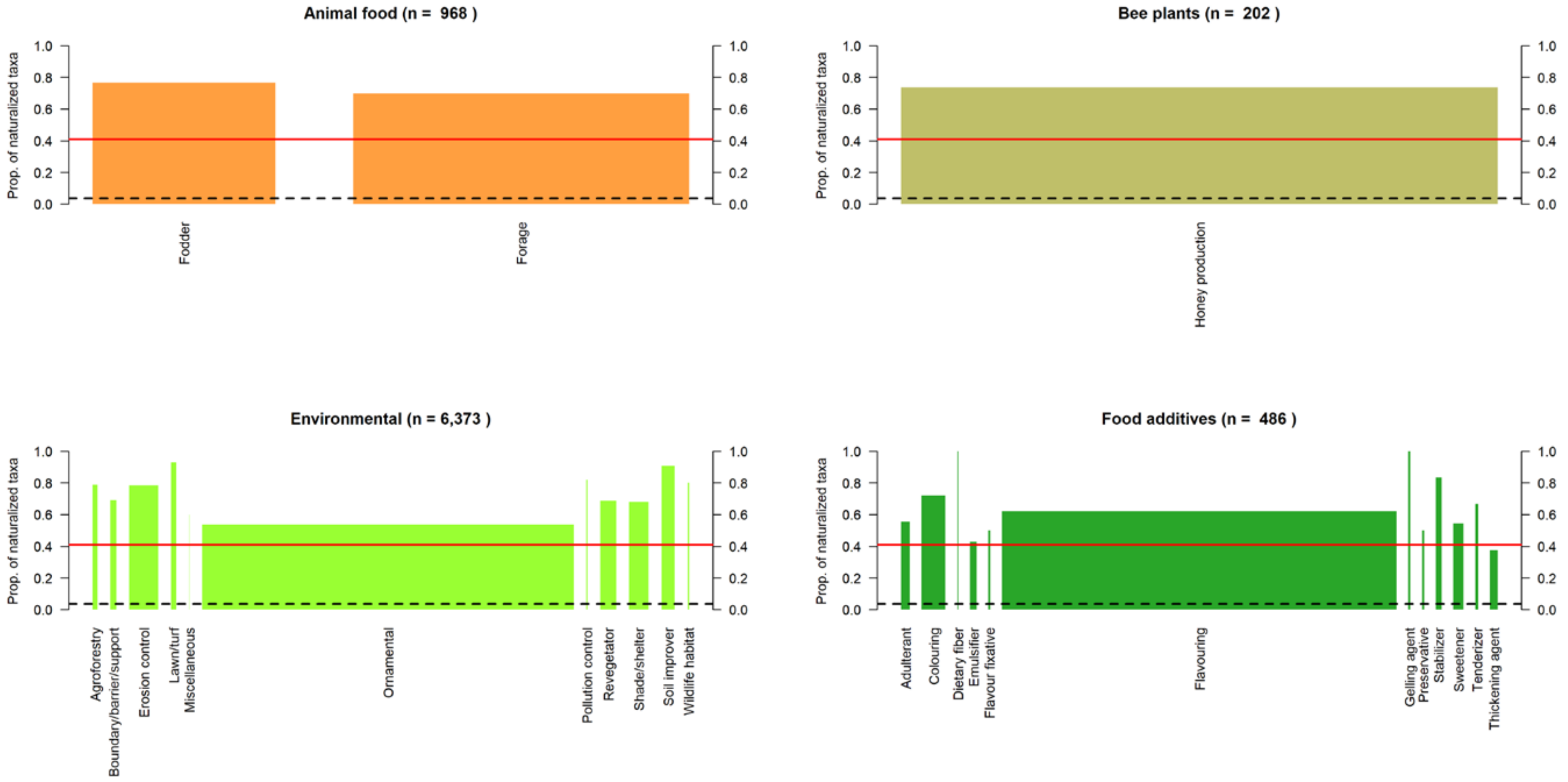

Supplementary Fig. 11 | The proportion of naturalized taxa within subcategories of each economic use category shown in Fig 1 of the main manuscript. The width of each bar is proportional to the number of taxa. As reference proportions, the red solid line in each spineplot indicates the proportion of naturalized taxa in the total WEP dataset, and the black dashed line indicates the proportion of naturalized taxa in the extant global seed-plant flora. For the categories environmental, gene sources and materials, the subcategories with <10 taxa were merged in a new subcategory ‘miscellaneous’. For each category, the cumulative number of taxa across the subcategories is given in brackets. Note, that this number can exceed the number of taxa listed for that category in Table 1, because some taxa are included in multiple subcategories.

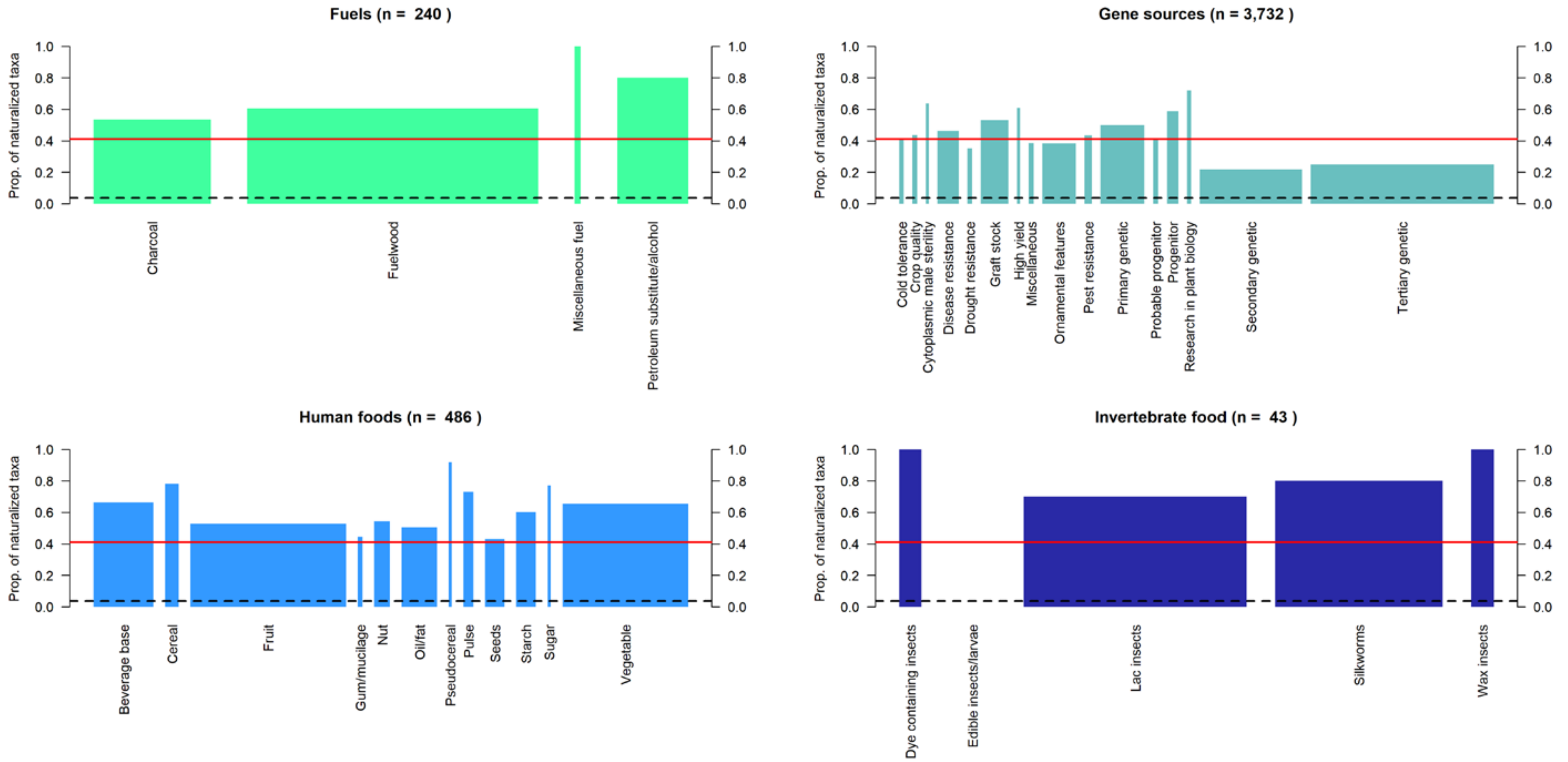

Supplementary Fig. 11 continued

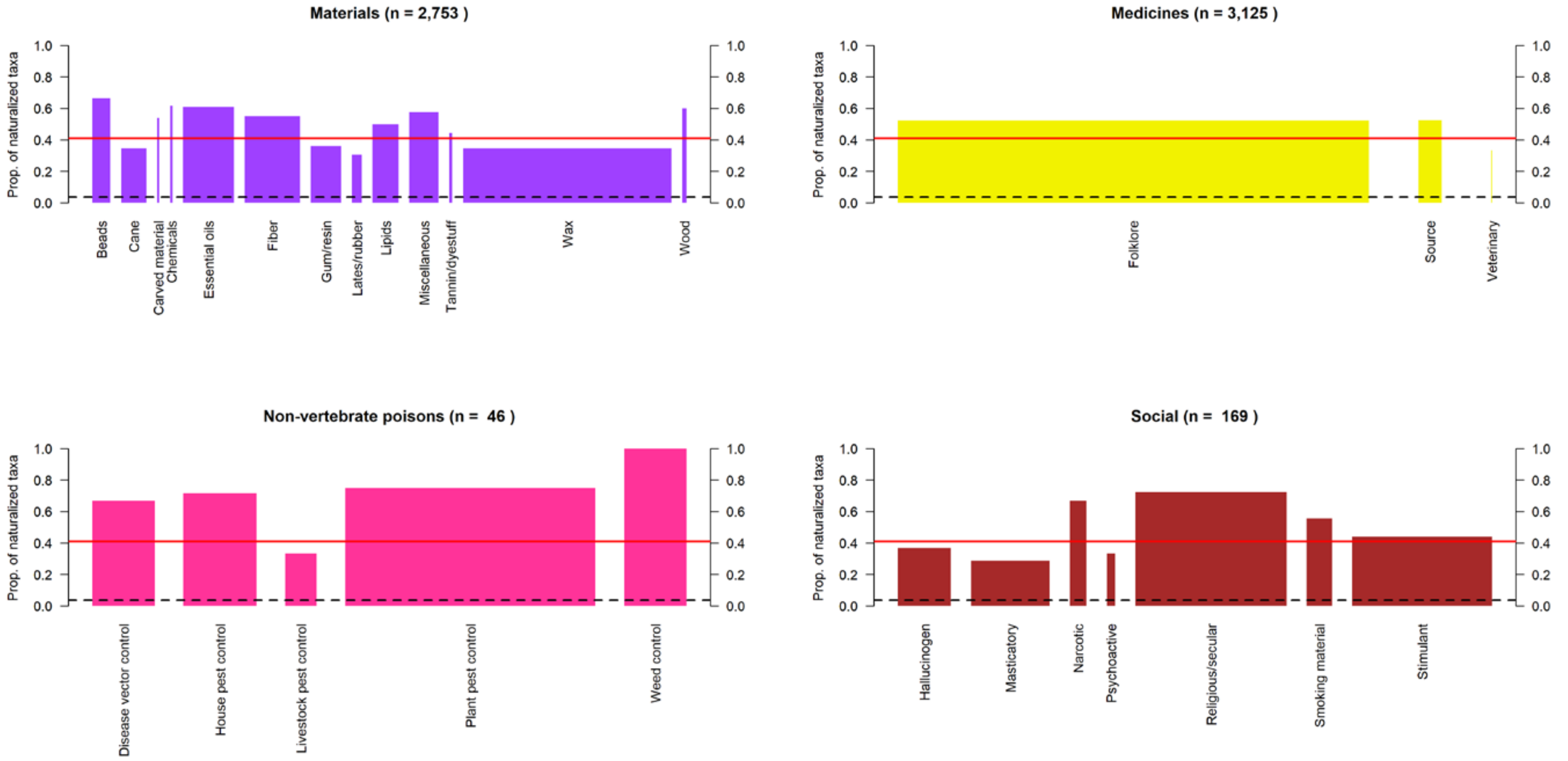

Supplementary Fig. 11 continued
