# Supplementary material for: Economic use of plants is key to their naturalization success
Source: Nat Commun. 2020 Jun 24;11:3201. doi: 10.1038/s41467-020-16982-3 (PMC7314777; doi:10.1038/s41467-020-16982-3)
Supplement: Supplementary file 3 — Description of Additional Supplementary Files [file 41467_2020_16982_MOESM3_ESM.pdf]

Description of Additional Supplementary Files

File Name: Supplementary Data 1

Description: The 861 regions used for making Fig. 7 with information on the number of naturalized species and the ones of those that have economic uses. The table also provides for each region the GloNAF codes and the latitude and longitude of the centroid.
