# Supplementary material for: Economic use of plants is key to their naturalization success
Source: Nat Commun. 2020 Jun 24;11:3201. doi: 10.1038/s41467-020-16982-3 (PMC7314777; doi:10.1038/s41467-020-16982-3)
Supplement: Supplementary file 5 — Reporting Summary [file 41467_2020_16982_MOESM5_ESM.pdf]

nature research

Corresponding author(s): Mark van Kleunen

Last updated by author(s): May 9, 2020

Reporting Summary

Nature Research wishes to improve the reproducibility of the work that we publish. This form provides structure for consistency and transparency in reporting. For further information on Nature Research policies, see Authors & Referees and the Editorial Policy Checklist .

Statistics

For all statistical analyses, confirm that the following items are present in the figure legend, table legend, main text, or Methods section.

n/a	Confirmed	
☐	☒	The exact sample size (*n*) for each experimental group/condition, given as a discrete number and unit of measurement
☒	☐	A statement on whether measurements were taken from distinct samples or whether the same sample was measured repeatedly
☐	☒	The statistical test(s) used AND whether they are one- or two-sided<br>*Only common tests should be described solely by name; describe more complex techniques in the Methods section.*
☐	☒	A description of all covariates tested
☒	☐	A description of any assumptions or corrections, such as tests of normality and adjustment for multiple comparisons
☐	☒	A full description of the statistical parameters including central tendency (e.g. means) or other basic estimates (e.g. regression coefficient) AND variation (e.g. standard deviation) or associated estimates of uncertainty (e.g. confidence intervals)
☐	☒	For null hypothesis testing, the test statistic (e.g. *F*, *t*, *r*) with confidence intervals, effect sizes, degrees of freedom and *P* value noted<br>*Give P values as exact values whenever suitable.*
☒	☐	For Bayesian analysis, information on the choice of priors and Markov chain Monte Carlo settings
☒	☐	For hierarchical and complex designs, identification of the appropriate level for tests and full reporting of outcomes
☐	☒	Estimates of effect sizes (e.g. Cohen's *d*, Pearson's *r*), indicating how they were calculated

Our web collection on statistics for biologists contains articles on many of the points above.

Software and code

Policy information about availability of computer code

Data collection	All collected data was first processed in Microsoft Excell 2016
Data analysis	All data was analysed using R version 3.6.1. We used the R packages Taxonstand (version 2.1), phytools (version 0.6-99), mblm (version 0.12.1), gam (version1.16.1)

For manuscripts utilizing custom algorithms or software that are central to the research but not yet described in published literature, software must be made available to editors/reviewers. We strongly encourage code deposition in a community repository (e.g. GitHub). See the Nature Research guidelines for submitting code & software for further information.

Data

Policy information about availability of data

All manuscripts must include a data availability statement. This statement should provide the following information, where applicable:

- Accession codes, unique identifiers, or web links for publicly available datasets
- A list of figures that have associated raw data
- A description of any restrictions on data availability

The databases that we used are all publicly available (World Economic Plants [WEP]: https://npgsweb.ars-grin.gov/gringlobal/taxon/taxonomysearcheco.aspx; Global Naturalized Alien Flora [GloNAF]: https://doi.org/10.1002/ecy.2542; World Checklist of Selected Plant Families [WCSP]: http://wcsp.science.kew.org/; The Plant List: http://www.theplantlist.org/, Smith and Brown phylogenetic tree: https://github.com/FePhyFoFum/big_seed_plant_trees). The data analysed during this study and the R syntax are available in "figshare" with the identifier doi:10.6084/m9.figshare.12278057.

Field-specific reporting

Please select the one below that is the best fit for your research. If you are not sure, read the appropriate sections before making your selection.

- [ ] Life sciences
- [ ] Behavioural & social sciences
- [x] Ecological, evolutionary & environmental sciences

For a reference copy of the document with all sections, see nature.com/documents/nr-reporting-summary-flat.pdf

Ecological, evolutionary & environmental sciences study design

All studies must disclose on these points even when the disclosure is negative.

Study description	We combined a global dataset on 11,685 plant taxa with economic uses (12 categories) with a global dataset on 12,010 naturalized plant taxa to analyse how economic use of plants relates to their naturalization success. For some of the analyses, we also considered the entire extant Spermatiophyta flora of 326,101 taxa. We analysed the continents of origin of the economic plants and the naturalized plants. Using a seed-plant phylogeny of the extant flora, we tested for phylogenetic signals in economic use and naturalization. In addition, we analysed for 861 regions around the world, the proportion of their naturalized alien flora that has an economic use.
Research sample	We used existing datasets. The global dataset of economic plants was extracted from the World Economic Plants (WEP) database (National Plant Germplasm System GRIN-GLOBAL; https://npgsweb.ars-grin.gov/gringlobal/taxon/taxonomysearcheco.aspx, accessed on 7 January 2016). The data on naturalization of the plants came from the Global Naturalized Alien Flora (van Kleunen et al. 2019; Ecology DOI: 10.1002/ecy.2542). Data on the extant Spermatophyte flora came from The Plant List (http://www.theplantlist.org/). The seed-plant phylogeny that we used is from Smith and Brown (2018; American Journal of Botany doi:10.1002/ajb2.1019), and the main data sources for the native ranges of the taxa were GRIN-GLOBAL and the World Checklist of Selected Plant Families (WCSP; http://wcsp.science.kew.org/).
Sampling strategy	We used all data available for taxa with accepted names in The Plant List (http://www.theplantlist.org/). As this is not an experimental study and we used all available data from the global databases, there was no need to predetermine sample sizes.
Data collection	The data was extracted by the authors and some of their lab technicians from the databases listed above in the Data section.
Timing and spatial scale	Data from the World Economic Plants (WEP) database was extracted on 7 January 2016. The original data in the Global Naturalized Flora database comes from regional naturalized plant inventories collected from 1918 to 2018. Data from World Checklist of Selected Plant Families (WCSP) had been extracted on 23 May 2014. Data from The Plant List had been extracted on 2 December 2019. There is no real rationale for the extraction dates; we simply happened to have extracted the data on those dates. These databases have not changed since then or only very little.
Data exclusions	To be able to align the different databases, all taxonomic names were standardized according to the names used in The Plant List (http://www.theplantlist.org/). Taxa that did not have accepted names in The Plant List were excluded.
Reproducibility	As we did not do an experiment, but analysed existing databases, we did not test for reproducibility of experiments.
Randomization	As we did not do an experiment, but analysed existing databases, we did not have to allocate samples randomly to groups.
Blinding	As we did not do an experiment, but analysed existing databases, blinding was not relevant to our study.

Did the study involve field work? [ ] Yes [x] No

Reporting for specific materials, systems and methods

We require information from authors about some types of materials, experimental systems and methods used in many studies. Here, indicate whether each material, system or method listed is relevant to your study. If you are not sure if a list item applies to your research, read the appropriate section before selecting a response.

Materials & experimental systems

n/a	Involved in the study	
x		Antibodies
x		Eukaryotic cell lines
x		Palaeontology
x		Animals and other organisms
x		Human research participants
x		Clinical data

Methods

n/a	Involved in the study	
x		ChIP-seq
x		Flow cytometry
x		MRI-based neuroimaging
